# Antimicrobial Activity of *Pediococcus pentosaceus* PMY2 Against Multidrug-Resistant Pathogens

**DOI:** 10.3390/antibiotics14040389

**Published:** 2025-04-08

**Authors:** Gyeong-Seon Yi, Xiangji Jin, Qiwen Zheng, Trang Thi Minh Nguyen, Su-Jin Yang, Tae-Hoo Yi

**Affiliations:** 1 Department of Convergent Biotechnology and Advanced Materials Engineering, Graduate School, Kyung Hee University, Yongin 17104, Republic of Korea; ks010924@khu.ac.kr; 2 Department of Dermatology, Graduate School, Kyung Hee University, Seoul 02447, Republic of Korea; hyanghe112@khu.ac.kr; 3 Graduate School of Biotechnology, Kyung Hee University, Yongin 17104, Republic of Korea; zhengqiwen@khu.ac.kr (Q.Z.); trangnguyen@khu.ac.kr (T.T.M.N.); stella@khu.ac.kr (S.-J.Y.)

**Keywords:** multidrug-resistant pathogens, MDR, lactic acid bacteria, *Pediococcus pentosaceus*, antimicrobial activity, anti-inflammatory effect

## Abstract

**Background/Objectives**: Multidrug-resistant (MDR) pathogens pose a critical challenge in infection treatment. *Pediococcus pentosaceus* (*P. pentosaceus*) is known for its antimicrobial activity; however, studies on its effects against MDR pathogens remain limited. This study aimed to evaluate the antimicrobial and biological activities of *P. pentosaceus* PMY2, isolated from fermented porcine colostrum yogurt, against MDR pathogens, including *Staphylococcus aureus* (*S. aureus*), *Pseudomonas aeruginosa* (*P. aeruginosa*), and *Escherichia coli* (*E. coli*). **Methods**: The antimicrobial, anti-inflammatory, and cytotoxic effects of *P. pentosaceus* PMY2 were evaluated in vitro. In addition, IL-6 and TNF-α levels were analyzed using an ELISA kit. **Results**: The MIC value against *S. aureus* KCTC 3881 and MRSA (CCARM 3089) was 0.31 mg/mL, while the MBC values were 0.63 mg/mL and 2.5 mg/mL, respectively. At MIC, biofilm formation was inhibited by 62.2% in *S. aureus* KCTC 3881 and by 51.5% in MRSA. CFS exhibited low cytotoxicity in RAW 264.7 macrophages and significantly reduced NO production, IL-6, and TNF-α levels, indicating strong anti-inflammatory effects. **Conclusions**: These findings suggest that *P. pentosaceus* PMY2 exhibited excellent antimicrobial and anti-inflammatory activity against MDR pathogens, demonstrating its potential as a natural antimicrobial agent. These results indicate that PMY2 CFS could be a promising candidate for addressing antibiotic resistance issues.

## 1. Introduction

The skin is the primary defense barrier, protecting the body from external pathogens [1]. However, when damaged, the compromised skin becomes vulnerable to infections caused by various microorganisms [2]. Bacterial pathogens, such as *Staphylococcus aureus* (*S. aureus*), *Pseudomonas aeruginosa* (*P. aeruginosa*), and *Escherichia coli* (*E. coli*), are major causative agents of wound infections and hospital-acquired infections, particularly in immunocompromised individuals [3]. Non-multidrug-resistant (MDR) pathogens can cause severe complications, including chronic infections and systemic inflammation, with MDR pathogens further exacerbating treatment challenges [4]. These pathogens proliferate at the wound site, triggering inflammatory responses and, in severe cases, leading to life-threatening complications such as sepsis [5]. Excessive inflammatory responses, characterized by elevated levels of pro-inflammatory cytokines, such as interleukin-6 (IL-6) and tumor necrosis factor-alpha (TNF-α), can exacerbate tissue damage and delay wound healing [6].

The rapid spread of MDR pathogens, including methicillin-resistant *S. aureus* (MRSA), MDR *P. aeruginosa*, and MDR *E. coli*, has become a critical challenge in clinical settings [7]. According to the World Health Organization (WHO), the misuse and overuse of antimicrobials in humans, animals, and agriculture have accelerated the emergence of resistant pathogens, complicating treatment and increasing healthcare burdens [8]. Current treatment strategies for bacterial infections include combination therapy, such as ciprofloxacin plus amikacin against *P. aeruginosa* and daptomycin plus rifampicin against MRSA, as well as antibiotic cycling and mutation inhibitors, all of which aim to suppress bacterial resistance [9]. However, the continuous evolution of resistant strains has increasingly limited existing antibiotics’ effectiveness. This issue requires alternative antimicrobial strategies as potential solutions, including natural antimicrobial agents.

Antimicrobial substances derived from lactic acid bacteria (LAB) have gained considerable attention as potential alternatives to conventional antibiotics [10]. LAB inhibit pathogenic bacteria through the production of bacteriocins, organic acids, and other bioactive metabolites [11]. Previous studies have demonstrated that LAB-derived bacteriocins exhibit strong antibacterial activity against various foodborne and clinical pathogens, including *S. aureus* and *E. coli* [12]. Additionally, LAB have been reported to exert antioxidant and anti-inflammatory effects, suggesting their potential in mitigating infection-related oxidative stress and inflammatory damage [13]. In particular, LAB-derived metabolites have been shown to modulate inflammatory responses by suppressing cytokine production, thereby reducing excessive immune activation in infected tissues [14]. While their applications in food preservation and gut microbiota modulation are well established, research on their effectiveness against MDR pathogens remains limited.

Among the various LAB species, *Pediococcus pentosaceus* (*P. pentosaceus*) has been highlighted for its antimicrobial activity against foodborne pathogens, including *Listeria monocytogenes*, *E. coli*, *Salmonella* spp., and *S. aureus*, as well as its antioxidant properties [15,16]. Commonly found in fermented foods, *P. pentosaceus* produces various bacteriocins, such as pediocin GS4, coagulin A, penocin A, and pentocin L, along with organic acids that effectively suppress the growth of pathogenic bacteria [15]. Although some studies have investigated the antimicrobial effects of *P. pentosaceus* strains isolated from milk-derived sources [17,18], their efficacy against MDR pathogens remains underexplored. Given the increasing prevalence of MDR pathogens, further investigation into the antimicrobial potential of *P. pentosaceus* is warranted to develop effective alternative treatment strategies.

This research aimed to evaluate the potential of *P. pentosaceus* PMY2 cell-free supernatant (CFS) as a natural antimicrobial agent by investigating its efficacy against MDR pathogens and its impact on immune responses. These findings provide a robust basis for further exploration of *P. pentosaceus* as a functional agent in skin health applications.

## 2. Results

### 2.1. Isolation and Identification of Lactic Acid Bacteria (LAB)

**Figure 1:**
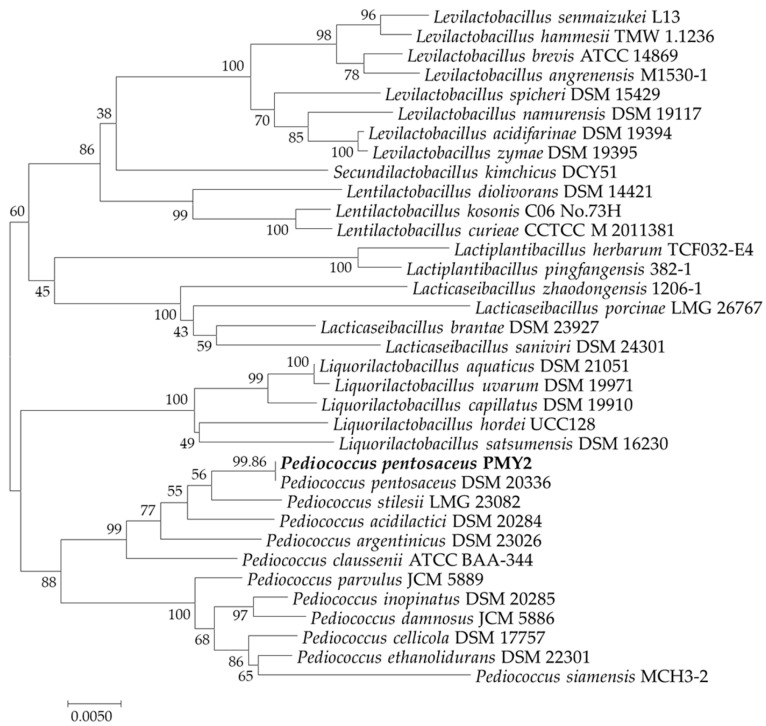
Neighbor-joining phylogenetic tree of *Pediococcus pentosaceus* PMY2 based on a comparative analysis of 16S rRNA gene sequences.

The PMY2 strain, identified through 16S rRNA gene sequencing, showed a 99.86% sequence similarity to *P. pentosaceus*. Based on this result, the strain was designated as *P. pentosaceus* PMY2 (Figure 1).

### 2.2. Comparison of the Antimicrobial Activity of P. pentosaceus PMY2 CFS and Its Fractions with Ampicillin

The antimicrobial activity of the CFS, distilled water (DW) fraction, and ethyl acetate (EA) fraction of *P. pentosaceus* PMY2 were evaluated using the disk diffusion assay (Table 1). Sterile 8 mm disks impregnated with each fraction at a concentration of 2.5 mg/disk were placed on agar plates inoculated with six indicator strains, alongside a control disk containing ampicillin (10 µg/disk; Sigma-Aldrich, St. Louis, MO, USA). No antibacterial activity was observed in the DW fraction for any of the tested strains. In contrast, both the CFS and EA fractions exhibited distinct zones of inhibition. Notably, the CFS demonstrated stronger antibacterial activity than the EA fraction across all indicator strains. Additionally, for antibiotic-resistant strains, ampicillin either showed no antimicrobial activity or weaker inhibition zones compared to the CFS. In particular, no inhibition zone was observed for *P. aeruginosa* KACC 10187.

### 2.3. MIC and MBC of Pediococcus pentosaceus PMY2 Against Pathogenic Bacteria

**Figure 2:**
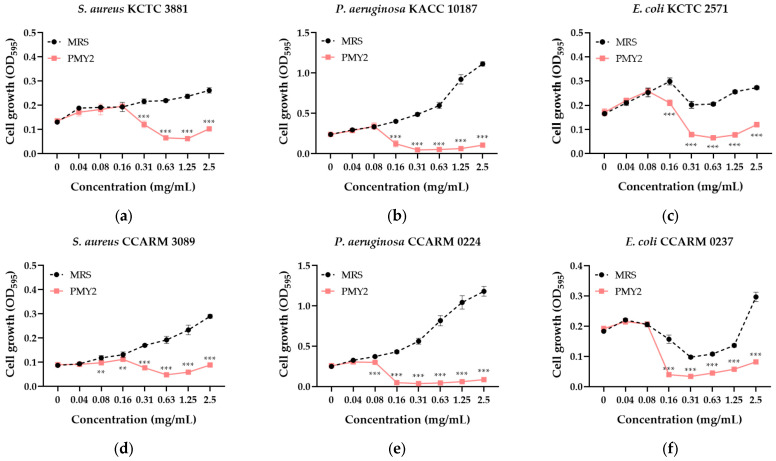
Antimicrobial activity of the cell-free supernatant from *Pediococcus pentosaceus* PMY2 determined by the broth microdilution assay. Statistical analysis was performed using Bonferroni’s multiple comparisons test (** *p* < 0.01, *** *p* < 0.001). (**a**) *S. aureus* KCTC 3881; (**b**) *P. aeruginosa* KACC 10187; (**c**) *E. coli* KCTC 2571; (**d**) *S. aureus* CCARM 3089; (**e**) *P. aeruginosa* CCARM 0224; (**f**) *E. coli* CCARM 0237.

The CFS of *P. pentosaceus* PMY2 effectively inhibited the growth of the tested pathogenic bacteria (Figure 2). In contrast, de Man, Rogosa, and Sharpe (MRS; Kisan Bio, Seoul, Republic of Korea) broth, used as a control, promoted the growth of *S. aureus* and *P. aeruginosa*, including their MDR strains, in a concentration-dependent manner. For *E. coli* and its MDR strain, bacterial growth initially increased with higher MRS broth concentrations, followed by a temporary decrease, and then increased again. The minimum inhibitory concentration (MIC) and minimum bactericidal concentration (MBC) values were evaluated and are summarized in Table 2. The MIC was determined to be 0.31 mg/mL for *S. aureus* and *S. aureus* CCARM 3089 (MRSA), and 0.16 mg/mL for *P. aeruginosa* and *E. coli*, including their MDR strains. The MBC was 0.63 mg/mL for *S. aureus*, *E. coli*, and MDR *E. coli* strains, 0.31 mg/mL for *P. aeruginosa* and its MDR strain, and 2.5 mg/mL for MRSA.

### 2.4. Biofilm Inhibition Activity of Pediococcus pentosaceus PMY2

**Figure 3:**
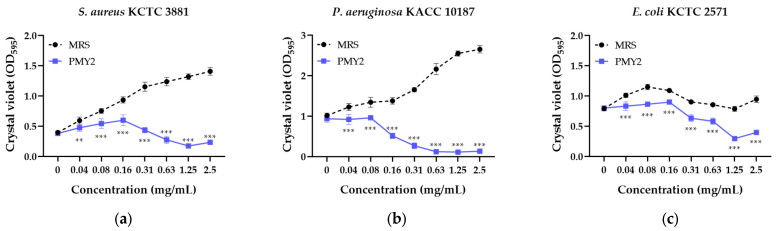

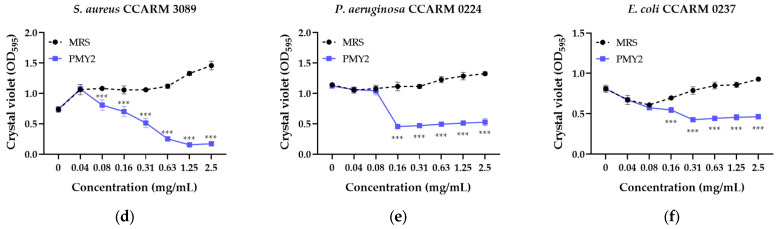
Biofilm inhibition activity of the cell-free supernatant from *Pediococcus pentosaceus* PMY2 using 0.01% crystal violet staining. Statistical analysis was performed using Bonferroni’s multiple comparisons test (** *p* < 0.01, *** *p* < 0.001). (**a**) *S. aureus* KCTC 3881; (**b**) *P. aeruginosa* KACC 10187; (**c**) *E. coli* KCTC 2571; (**d**) *S. aureus* CCARM 3089; (**e**) *P. aeruginosa* CCARM 0224; (**f**) *E. coli* CCARM 0237.

The biofilm inhibition assay demonstrated that the CFS of *P. pentosaceus* PMY2 significantly reduced biofilm formation in all tested strains (Figure 3). Consistent with the results of the broth microdilution assay, most strains exhibited a concentration-dependent increase in biofilm formation in the presence of MRS broth. However, *E. coli* and its MDR strain showed an initial increase in biofilm formation with higher MRS broth concentrations, followed by a temporary decrease, and then an increase again. Biofilm inhibition was observed at concentrations lower than the MIC for *E. coli* and MRSA, whereas for the other tested strains, inhibition was detected at concentrations equivalent to the MIC. Specifically, *S. aureus* exhibited biofilm inhibition rates of approximately 3.9%, 19.9%, 27.7%, 35.8%, 62.2%, 77.5%, 86.6%, and 83.2% at 0.04, 0.08, 0.16, 0.31, 0.63, 1.25, and 2.5 mg/mL, respectively. For MRSA, inhibition rates were 25.2%, 33.6%, 51.5%, 77.5%, 88.3%, and 88.2% at 0.08, 0.16, 0.31, 0.63, 1.25, and 2.5 mg/mL, respectively. These results indicate that the antimicrobial activity of CFS not only suppresses planktonic bacterial growth but also effectively inhibits biofilm formation, with strain-dependent variations in sensitivity.

### 2.5. Protein Profile and Antimicrobial Activity of the EA Fraction from Pediococcus pentosaceus PMY2

**Figure 4:**
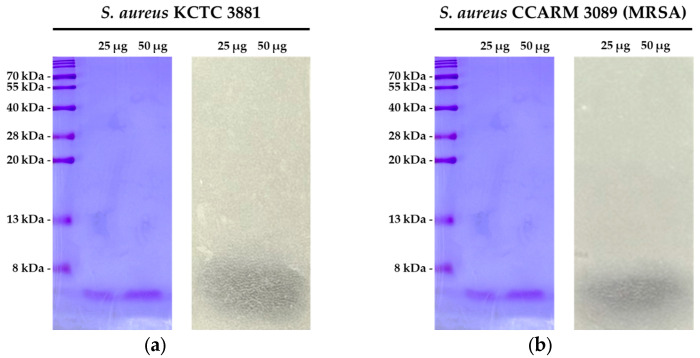
SDS-PAGE and overlay agar assay results of the EA fraction of *Pediococcus pentosaceus* PMY2. (**a**) SDS-PAGE analysis of the EA fraction, followed by an overlay agar assay against *S. aureus* KCTC 3881, demonstrating the antimicrobial activity of the EA fraction; (**b**) SDS-PAGE analysis of the EA fraction, followed by an overlay agar assay against *S. aureus* CCARM 3089 (MRSA), confirming its antimicrobial activity.

SDS-PAGE analysis of the EA fraction of the CFS from strain PMY2 revealed the size of the antimicrobial substances. A comparison with the protein marker indicated that the antimicrobial substances in the EA fraction were smaller than 8 kDa. The overlay agar assay confirmed the antimicrobial activity of the proteins in this fraction, as indicated by the formation of a clear zone at the corresponding protein size (Figure 4a,b). These results suggest that the antimicrobial activity observed in the EA fraction of PMY2 is likely attributed to these small proteins.

### 2.6. Antioxidant Activity Assays of Pediococcus pentosaceus PMY2

**Figure 5:**
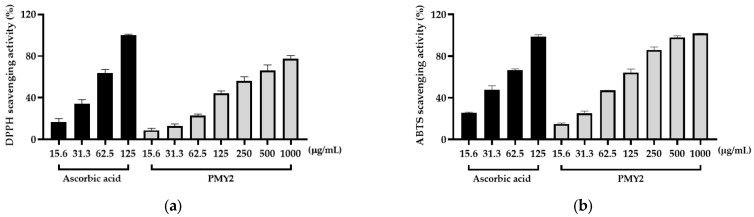
Antioxidant activity of the cell-free supernatant from *Pediococcus pentosaceus* PMY2. (**a**) DPPH radical scavenging activity; (**b**) ABTS radical scavenging activity.

The antioxidant activity of PMY2 CFS was evaluated using DPPH and ABTS radical scavenging assays, with ascorbic acid as a positive control. In the DPPH assay, ascorbic acid showed nearly 100% scavenging activity at 125 µg/mL, with 63.6%, 34.6%, and 16.7% activity at 62.5, 31.3, and 15.6 µg/mL, respectively (Figure 5a). PMY2 exhibited 77.5% scavenging activity at 1000 µg/mL, decreasing to 66.3%, 56%, 23.1%, 12.8%, and 8.8% at 500, 250, 62.5, 31.3, and 15.6 µg/mL, respectively. The IC_50_ value of PMY2 for the DPPH assay was 2.301 μg/mL, which was higher than that of ascorbic acid (IC_50_: 1.608 μg/mL), indicating moderate antioxidant activity.

In the ABTS assay, ascorbic acid achieved 98.5% scavenging at 125 µg/mL, with 66.5%, 47.7%, and 25.6% at 62.5, 31.3, and 15.6 µg/mL, respectively (Figure 5b). PMY2 demonstrated a concentration-dependent scavenging effect, reaching nearly 100% at 1000 µg/mL, with 97.8%, 85.8%, 64.1%, 47.2%, 25.1%, and 15% at 500, 250, 125, 62.5, 31.3, and 15.6 µg/mL, respectively. The IC_50_ value of PMY2 for the ABTS assay was 1.816 μg/mL compared to 1.495 μg/mL for ascorbic acid. These results indicate that PMY2 exhibits significant scavenging activity in the ABTS assay, particularly at higher concentrations, with moderate antioxidant activity observed in the DPPH assay.

### 2.7. Anti-Inflammatory Effects of P. pentosaceus PMY2 CFS in LPS-Stimulated RAW 264.7 Cells

The anti-inflammatory effects of PMY2 CFS were evaluated by measuring NO production in LPS-stimulated RAW 264.7 cells. When comparing the untreated control group to the LPS-treated group, NO production increased by approximately 344%. However, when PMY2 CFS was administered at concentrations of 12.5, 25, 50, and 100 μg/mL, NO production was reduced by approximately 47.4%, 59.9%, 83.2%, and 104%, respectively, demonstrating a concentration-dependent inhibition of NO production (Figure 5a). The highest inhibition was observed at 100 μg/mL, indicating strong anti-inflammatory potential (*p* < 0.001).

To confirm that the reduction in NO production was not due to cytotoxic effects, an MTT assay was conducted following the NO assay. As shown in Figure 5b, cell viability in the LPS-treated group was approximately 91.7%. In the groups treated with PMY2 CFS at 12.5, 25, 50, and 100 μg/mL, cell viability was recorded as approximately 102.6%, 94.9%, 93.4%, and 94.6%, respectively. These results indicate that PMY2 CFS did not significantly affect cell viability, supporting the conclusion that its anti-inflammatory effects were not attributed to cytotoxicity.

### 2.8. Inhibitory Effects of PMY2 CFS on LPS-Induced IL-6 and TNF-α Secretion in RAW 264.7 Cells

Treatment of RAW 264.7 cells with 1 μg/mL lipopolysaccharide (LPS) significantly increased the secretion of IL-6 and TNF-α by approximately 138.9% and 107%, respectively. The CFS of PMY2 inhibited the production of these cytokines in a dose-dependent manner. For IL-6, the positive control (10 μM ampicillin) reduced its secretion by approximately 78.4%, whereas treatment with CFS at 25, 50, and 100 μg/mL resulted in reductions of 55.3%, 67.6%, and 88.1%, respectively. For TNF-α, the positive control exhibited an inhibitory effect of approximately 68.3%, while CFS at 25, 50, and 100 μg/mL reduced TNF-α levels by 40.5%, 58.2%, and 72.4%, respectively. Although the inhibitory effect of CFS was lower than that of 10 μM ampicillin at lower concentrations, at 100 μg/mL, the CFS exhibited superior inhibitory activity compared to the positive control.

## 3. Discussion

In this study, the CFS of *Pediococcus pentosaceus* PMY2 was evaluated for its antimicrobial, antibiofilm, anti-inflammatory, antioxidant, and cytotoxic effects, particularly against *S. aureus*, *P. aeruginosa*, *E. coli*, and their MDR strains. These pathogens pose significant treatment challenges due to their resistance mechanisms [19], promoting persistent infections and inflammation that delay wound healing [20,21]. *S. aureus* bacteremia (SAB) [22], biofilm-forming *P. aeruginosa* [23], and endotoxin-producing *E. coli* [24] exacerbate inflammatory responses by inducing excessive IL-6 and TNF-α secretion [25], contributing to severe tissue damage and systemic complications [26,27,28]. Given these infection dynamics, PMY2 CFS not only inhibits bacterial growth and biofilm formation but also regulates cytokine secretion, mitigating inflammation and oxidative stress [29].

The CFS of *P. pentosaceus* PMY2 exhibited strong antimicrobial activity against *S. aureus*, *P. aeruginosa*, *E. coli*, and their MDR strains, as confirmed through various assays (Table 2, Figure 2 and Figure 3). Notably, the control antibiotic ampicillin failed to inhibit MDR strains MRSA and *P. aeruginosa* CCARM 0224, showing no inhibition zones. In particular, no inhibition zone was observed for *P. aeruginosa* KACC 10187, which is consistent with previous findings that *P. aeruginosa* naturally expresses β-lactamase (especially AmpC β-lactamase), allowing it to readily hydrolyze ampicillin [30]. In contrast, CFS exhibited superior antimicrobial activity not only against these strains but also against *E. coli* CCARM 0237, demonstrating greater efficacy than ampicillin. This suggests that CFS exerts its antimicrobial effects through distinct mechanisms from conventional antibiotics [31]. Additionally, CFS effectively suppressed biofilm formation, even at sub-MIC concentrations for certain strains, with *E. coli* KCTC 2571 and MRSA showing inhibition at lower concentrations than their MICs. The antimicrobial activity appears to result from synergistic interactions among multiple bioactive compounds, including small peptides or bacteriocins, rather than a single dominant agent [32]. The low activity of the DW fraction suggests that key antimicrobial components are not highly water-soluble, while the EA fraction retained partial activity, indicating the presence of hydrophobic compounds [33]. Given that CFS exhibited the strongest antibacterial and antibiofilm effects, its potential as an alternative strategy for combating MDR infections is further supported. Future studies should characterize these active compounds and elucidate their mechanisms, particularly in relation to MDR pathogen inhibition and biofilm disruption. In particular, further experimental validation is required to confirm the antimicrobial activity of the proteins identified in the EA fraction. Mass spectrometry and affinity tag purification, followed by functional assays, would provide a more precise identification and characterization of these proteins, contributing to a clearer understanding of the molecular mechanisms underlying their antimicrobial effects.

**Figure 6:**
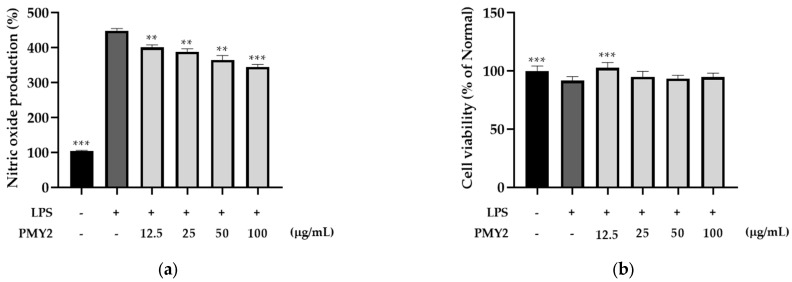
Effects of the cell-free supernatant from *Pediococcus pentosaceus* PMY2 on RAW 264.7 cells. (**a**) Nitric oxide (NO) production assay conducted to evaluate the anti-inflammatory effects of the cell-free supernatant. RAW 264.7 cells were induced with LPS and treated with varying concentrations of the supernatant. NO production was measured using the Griess reagent assay to assess the supernatant’s effect on inflammation; (**b**) MTT assay performed following the NO production assay to assess cell viability and confirm that the observed NO inhibition was not due to cytotoxic effects. Statistical analysis was carried out using Brown–Forsythe and Welch ANOVA followed by Dunnett’s T3 multiple comparisons test (** *p* < 0.01, *** *p* < 0.001).

Beyond its antimicrobial properties, PMY2 CFS exhibited notable antioxidant activity, particularly in the ABTS assay at higher concentrations (Figure 5b). Although its efficacy was slightly lower than that of ascorbic acid, its concentration-dependent radical-scavenging ability suggests its potential in mitigating oxidative stress, a key factor in inflammation, aging, and chronic wounds [34]. Consistently, in LPS-stimulated RAW 264.7 macrophages, PMY2 CFS significantly reduced NO production in a concentration-dependent manner (Figure 6a), indicating its potential role in suppressing oxidative stress-induced inflammation [29]. Furthermore, cytotoxicity assessments confirmed that PMY2 CFS exhibited minimal toxicity in RAW 264.7 macrophages (Figure 6b), supporting its potential as a safe bioactive agent.

**Figure 7:**
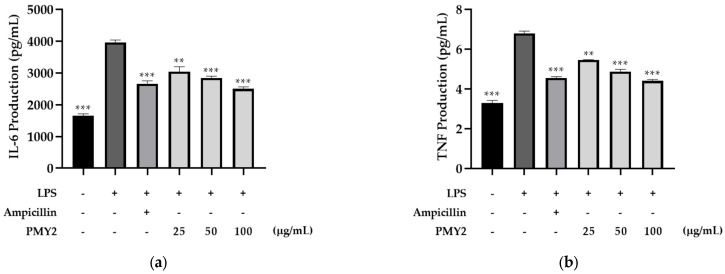
Inhibitory effects of the cell-free supernatant from *Pediococcus pentosaceus* PMY2 on LPS-induced Mouse IL-6 and Mouse TNF-α secretion in RAW 264.7 cells. (**a**) IL-6 levels; (**b**) TNF-α levels. Cells were treated with PMY2 CFS at 25, 50, and 100 μg/mL for 1 h, followed by LPS (1 μg/mL) stimulation for 24 h. The positive control was treated with 10 μM ampicillin. Statistical analysis was performed using Brown–Forsythe and Welch ANOVA followed by Dunnett’s T3 multiple comparisons test (** *p* < 0.01; *** *p* < 0.001).

Furthermore, PMY2 CFS demonstrated notable anti-inflammatory properties by downregulating pro-inflammatory cytokines. Specifically, it suppressed LPS-induced IL-6 and TNF-α secretion in a concentration-dependent manner (Figure 7a,b). Notably, IL-6 and TNF-α levels were lower in the group treated with 100 μg/mL of PMY2 CFS compared to the group treated with 10 μM of ampicillin. This suggests that PMY2 CFS may effectively mitigate excessive inflammatory responses through immunomodulation rather than solely through antimicrobial activity. Since uncontrolled cytokine secretion is a key contributor to chronic inflammation and tissue damage [35], this regulatory effect highlights the potential therapeutic applications of PMY2 CFS. While its antimicrobial activity effectively targets pathogenic bacteria, including MDR strains, its ability to modulate cytokine levels suggests its potential as a direct immunomodulatory mechanism and not just a bacterial suppressant [14]. This dual action of PMY2 CFS, combining antimicrobial and anti-inflammatory properties, may offer advantages in managing infections with associated inflammatory complications. Further studies are needed to clarify its precise mechanisms and evaluate its efficacy in in vivo models to support its potential clinical applications.

Overall, PMY2 CFS demonstrated its potential as a natural antimicrobial agent with both antibacterial and anti-inflammatory activities against MDR pathogens. It effectively inhibited the growth and biofilm formation of *S. aureus*, *P. aeruginosa*, and *E. coli*, including their MDR strains, while also exhibiting immunomodulatory effects by suppressing the production of pro-inflammatory cytokines. Although higher concentrations were required compared to the conventional antibiotic ampicillin, its natural origin highlights its potential as an alternative therapeutic strategy to combat antibiotic resistance.

However, this study has some limitations. The anti-inflammatory effects were evaluated with a focus on IL-6 and TNF-α, and the experiments were conducted solely using RAW 264.7 macrophages. Further studies should include HaCaT keratinocytes or fibroblasts to provide a more comprehensive understanding of the anti-inflammatory mechanisms of PMY2 CFS. Additionally, as this study was conducted in vitro, in vivo experiments and clinical investigations are required to further validate its therapeutic efficacy. Moreover, the long-term stability and shelf-life of the antimicrobial compounds produced by *P. pentosaceus* PMY2 have not been evaluated, which are crucial factors for practical applications. Future studies should investigate the stability of PMY2-derived bioactive compounds under various storage conditions and determine their long-term efficacy. Taken together, these findings suggest that future research should focus on elucidating the precise mechanism of action of PMY2 CFS and assessing its therapeutic potential in in vivo models to support its clinical application.

## 4. Materials and Methods

### 4.1. Bacterial Strain Cultivation

The bacterial strains used in this study included *S. aureus* and *E. coli*, which were obtained from the Korean Collection for Type Cultures (KCTC), and *P. aeruginosa*, which was obtained from the Korean Agricultural Culture Collection (KACC). Additionally, MDR strains of *S. aureus*, *P. aeruginosa*, and *E. coli* were obtained from the Culture Collection of Antibiotic-Resistant Microbes (CCARM). All six strains were incubated at 30 °C. A single colony of each strain was inoculated into nutrient broth (NB; Kisan Bio Co., Nonsan, Republic of Korea) and cultured for 24 h. The cultures were then transferred to fresh NB broth and incubated for an additional 24 h before use.

### 4.2. Isolation and Identification of Lactic Acid Bacteria

To isolate LAB, 1 mL of porcine colostrum was anaerobically fermented at 30 °C for three days. The fermented sample was then centrifuged at 14,000 rpm for 20 min at 4 °C to obtain the whey supernatant. A 1 μL loop of the supernatant was streaked onto MRS agar (Kisan Bio Co., Republic of Korea) and incubated at 30 °C for 24 h. Eight distinct colonies were selected and transferred to bromocresol purple (BCP) agar (Eiken Chemical Co., Ltd., Tokyo, Japan), where they turned yellow within 12 h, indicating lactic acid production. Disk diffusion screening identified the most active strain as PMY2. The strain was identified by 16S rRNA gene sequencing using the primers 1492R and 27F. Sequencing was performed by Biofact (Daejeon, Republic of Korea), and sequence similarity analysis was conducted using the EZBioCloud database— https://www.ezbiocloud.net/ (accessed on 22 April 2024).

### 4.3. Preparation of Cell-Free Supernatant (CFS) and Its Fractions

A single colony grown on MRS agar was inoculated into 5 mL of MRS broth and incubated at 30 °C for 48 h. The culture was centrifuged at 3500 rpm for 15 min at 4 °C, and the supernatant was filtered through a 0.22 µm membrane filter to obtain the CFS. For fractionation, the culture was scaled up by inoculating a 24 h pre-culture (5 mL of MRS broth) into 500 mL of MRS broth, followed by incubation under the same conditions. After incubation, the culture was extracted three times with EA in a 1:1 (*v*/*v*) ratio. The EA and DW fractions were then concentrated by solvent removal under reduced pressure using a rotary evaporator.

### 4.4. Antimicrobial Activity via Disk Diffusion Assay

The antimicrobial activity of *P. pentosaceus* PMY2 CFS and its fractions was evaluated using the disk diffusion assay. EA and DW fractions were obtained from the culture supernatant and applied onto 8 mm sterile disks at a concentration of 2.5 mg/disk, followed by thorough drying. The prepared disks were placed on agar plates inoculated with six indicator strains adjusted to 1.5 × 10^8^ CFU/mL using sterile cotton swabs. A control disk containing ampicillin (10 µg/disk) was included for comparison. The plates were incubated at 30 °C overnight, and the diameters of the inhibition zones (mm) were measured to assess antimicrobial activity.

### 4.5. MIC and MBC Determination by Broth Microdilution Assay

The antimicrobial activity, including MIC and MBC, of the CFS from *P. pentosaceus* PMY2 was evaluated using the broth microdilution method. The prepared CFS was diluted to concentrations of 2.5, 1.25, 0.63, 0.31, 0.16, 0.08, 0.04, and 0 mg/mL, then mixed with pathogenic bacteria, including MDR strains, at a 1:1 ratio, and dispensed into a 96-well plate. The mixtures were incubated at 37 °C for 24 h, and bacterial growth inhibition was determined by measuring OD values at 595 nm using a microplate reader (Molecular Devices, San Francisco, CA, USA). The lowest concentration that inhibited visible growth was defined as the MIC. To determine the MBC, aliquots from each concentration were streaked onto Mueller–Hinton agar (MHA; Difco, Franklin Lakes, NJ, USA) and incubated at 30 °C for 24 h. The lowest concentration with no colony formation was defined as the MBC. MRS broth was used as a negative control.

### 4.6. Biofilm Inhibition Assay

The biofilm inhibitory activity of the CFS from *P. pentosaceus* PMY2 was assessed using the crystal violet staining method. The CFS was diluted to final concentrations of 2.5, 1.25, 0.63, 0.31, 0.16, 0.08, 0.04, and 0 mg/mL. Each dilution was mixed at a 1:1 ratio with the culture medium of pathogenic bacteria, including MDR strains, and dispensed into a 96-well plate. The plate was incubated at 37 °C for 24 h to allow biofilm formation. Following incubation, the wells were gently washed twice with DW to remove non-adherent bacteria. The remaining biofilm was stained with 0.01% crystal violet solution for 15 min at room temperature. Excess stain was removed by washing with DW, and the plate was air-dried. The bound crystal violet was solubilized with 33% acetic acid, and absorbance was measured at 595 nm using a microplate reader.

### 4.7. SDS-PAGE and Overlay Agar Assay

SDS-PAGE was performed to analyze the protein composition of the EA fraction from PMY2. A 15% polyacrylamide gel was used, and molecular weight markers ranging from 8 to 200 kDa were included. Electrophoresis was conducted at 80 V during stacking and 120 V for protein separation. After electrophoresis, all gels were thoroughly washed to remove residual SDS. One gel was stained with 0.01% crystal violet to clearly visualize protein sizes, while the other two gels were used for overlay agar assays. The washed gels were overlaid with overlay agar containing *S. aureus* and MRSA, respectively, followed by incubation to assess antimicrobial activity through clear zone formation.

### 4.8. Antioxidant Activity Assays

The antioxidant activity of PMY2 CFS was assessed using DPPH and ABTS assays. Serial dilutions of CFS (15.6, 31.3, 62.5, 125, 250, 500, and 1000 μg/mL) were prepared, with ascorbic acid used as a positive control at concentrations of 15.6, 31.3, 62.5, and 125 μg/mL. For the DPPH assay, samples were incubated for 30 min, while for the ABTS assay, incubation lasted 15 min. Absorbance was measured at 595 nm DPPH and 620 nm ABTS using a microplate reader. Antioxidant activity was calculated as a percentage of the control.

### 4.9. Cell Culture

The murine macrophage cell line RAW 264.7 was sourced from the Korean Cell Line Bank (KCLB, Seoul, Republic of Korea). These cells were cultured in Dulbecco’s Modified Eagle Medium (DMEM; Gibco, Carlsbad, CA, USA) supplemented with 10% heat-inactivated fetal bovine serum (FBS; Gibco) and 100 U/mL penicillin. The incubation conditions were maintained at 37 °C with 5% CO_2_ in a humidified environment.

### 4.10. Anti-Inflammatory Effects via NO Production Assay in RAW 264.7 Cells

The anti-inflammatory effects of PMY2 CFS were evaluated by measuring NO production in RAW 264.7 cells. Cells were stimulated with LPS to induce NO production and treated with various concentrations of CFS (12.5, 25, 50, and 100 μg/mL). NO levels were quantified using the Griess assay, and results were expressed as a percentage relative to the negative control. Cytotoxicity was assessed in parallel to ensure non-toxic conditions.

### 4.11. Quantification of IL-6 and TNF-α in RAW 264.7 Cells by ELISA

The expression levels of Mouse IL-6 and Mouse TNF-α in RAW 264.7 cells were analyzed using ELISA kits (BD Biosciences, San Jose, CA, USA). RAW 264.7 cells were treated with the CFS derived from PMY2 at concentrations of 25, 50, and 100 µg/mL for 1 h, followed by stimulation with LPS. After 24 h of incubation, the culture supernatants were collected. The negative control consisted of cells cultured in DMEM, while the positive control was prepared by treating cells with 10 µM ampicillin for 1 h before LPS stimulation. The collected supernatants were analyzed according to the protocol provided by the manufacturer.

### 4.12. Statistical Analysis

All statistical analyses were performed using GraphPad Prism 8 (GraphPad Software, San Diego, CA, USA). Data are presented as mean ± standard deviation (SD). Statistical significance was set at *p* < 0.05, with additional significance levels indicated as *p* < 0.01 and *p* < 0.001. The statistical tests used included Bonferroni’s multiple comparisons test, Brown–Forsythe and Welch ANOVA, followed by Dunnett’s T3 multiple comparisons test as appropriate for each dataset.

## 5. Conclusions

This study demonstrates the antimicrobial, antibiofilm, antioxidant, and anti-inflammatory potential of *P. pentosaceus* PMY2 CFS, particularly against both common pathogenic bacteria and MDR pathogens. PMY2 CFS exhibited significant antimicrobial activity through mechanisms distinct from conventional antibiotics and effectively inhibited biofilm formation. Its antioxidant and anti-inflammatory properties further highlight its potential in mitigating oxidative stress and inflammation. While these findings suggest promising biomedical applications, further studies are needed to characterize the bioactive compounds, evaluate in vivo efficacy, and explore synergistic effects with antibiotics for enhanced therapeutic strategies.

## Figures and Tables

**Table 1:** Zone of inhibition (mm) produced by the cell-free supernatant, its fractions (distilled water and ethyl acetate), and ampicillin against pathogenic bacteria.

Indicators	Zone of Inhibition (mm)			
	Ampicillin 10 μg/disk	Distilled Water Fraction 2.5 mg/disk	Cell-Free Supernatant 2.5 mg/disk	Ethyl Acetate Fraction 2.5 mg/disk
*S. aureus* KCTC 3881	36	nd	17	15
*P. aeruginosa* KACC 10187	nd	nd	16	15
*E. coli* KCTC 2571	16	nd	15	14
*S. aureus* CCARM 3089	nd	nd	15	14
*P. aeruginosa* CCARM 0224	nd	nd	17	16
*E. coli* CCARM 0237	14	nd	15	14

nd: Not detected.

**Table 2:** Minimum inhibitory concentration (MIC) and minimum bactericidal concentration (MBC) of *Pediococcus pentosaceus* PMY2 cell-free supernatant against pathogenic bacteria.

Indicators	MIC (mg/mL)	MBC (mg/mL)
*S. aureus* KCTC 3881	0.31	0.63
*P. aeruginosa* KACC 10187	0.16	0.31
*E. coli* KCTC 2571	0.16	0.63
*S. aureus* CCARM 3089	0.31	2.5
*P. aeruginosa* CCARM 0224	0.16	0.31
*E. coli* CCARM 0237	0.16	0.63

## Data Availability

Data are contained within the article.
